# Comparative Evaluation of Two Different Fiber-Reinforced Composite Materials in Class 1 Post-Endodontic Restorations in Molars—A Randomized Clinical Study

**DOI:** 10.3390/ma15217858

**Published:** 2022-11-07

**Authors:** Suwidhi Ranka, Ajay Singh Rao, Unnati Shah, Dikshit Solanki, Ajinkya M. Pawar, Rodolfo Reda, Alessio Zanza, Luca Testarelli

**Affiliations:** 1 Department of Conservative Dentistry and Endodontics, KM Shah Dental College & Hospital, Sumandeep Vidyapeeth, Vadodara 391760, India; 2 Department of Conservative Dentistry and Endodontic, Nair Hospital Dental College, Mumbai 400034, India; 3 Department of Oral and Maxillofacial Sciences, Sapienza University of Rome, 00161 Rome, Italy

**Keywords:** endodontically treated tooth, everX Flow, everX Posterior, G-aenial universal injectable, solareX

## Abstract

This study aimed to evaluate and compare two different fiber-reinforced composite materials in class I post-endodontic restoration in molars. A total of 50 patients were randomly assigned into two groups (*n* = 25 for each group); group A: everX Posterior (packable composite) with a top layer of solareX (nano-hybrid composite) and group B: everX Flow (flowable composite) with a top layer of G-aenial universal injectable (flowable composite). Patients were evaluated immediately after the procedure (baseline), at 6 months, and at 1 year time intervals based on the modified USPHS criteria. The statistical analysis using a chi-square test showed no statistically significant difference in the clinical performance of group A and group B. Clinical performance of the combination of everX Flow with overlying G-aenial universal injectable composite proved to be comparable with everX Posterior with overlying solareX composite as post-endodontic restorations in class I lesions in permanent molars.

## 1. Introduction

Restoration of endodontically treated teeth is one of the most challenging parts of operative dentistry [1]. Fracture susceptibility of root canal-treated teeth is greater than vital teeth because of extensive loss of tooth structure due to caries and cleaning, and shaping of the root canal [2,3].

There are several factors responsible for the fracture of root canal-treated teeth such as the chemical nature of irrigants and intra-canal medicaments, non-iatrogenic factors like history of recurrent pathology, anatomical position of teeth, and effect of aging of dental tissues [2].

Restoration of root-filled teeth is of great significance in providing resistance to fracture [4]. According to classic literature, a full-coverage restoration is considered the routine treatment option as post-endodontic restoration, especially in molars. However, there are situations where we cannot go for full coverage restorations such as if a tooth has not fully erupted, or in the situation where the tooth undergoing root canal therapy needs to be kept for observation for a longer duration or where the evaluation of the healing of periapical lesion is needed. In such cases, to prevent the failure of the root canal, a simple, quick, high-strength, direct restorative material is necessary [5]. Composite restorations are most commonly used direct restorative materials but certain problems still exist with them such as secondary caries, fracture, marginal deficiencies, postoperative sensitivity, poor wear resistance [6,7], and polymerization shrinkage (which could range from 2.6% to 7.1%) [8]. 

The most important factor for the success of post-endodontic restorations is the selection of appropriate restorative materials and techniques to compensate for the loss of coronal tooth structure [9,10]. The conventional methods of post-endodontic restorations include the usage of cast restorations, full-coverage crowns, etc. However, most of these methods have led to the fracture of the root and/or crown structure as a result of a weakening of the tooth structure [11].

To date, composite resins are not regularly used for extensive restorations or in high-stress-bearing areas, despite many advancements in material science because of their low fracture resistance and polymerization shrinkage leading to the formation of micro-cracks in the tooth structure [12]. The introduction of fiber-reinforced composites (FRC) has provided a considerable opportunity to modify behavior and enhance the response of existing conventional materials. The reinforcement of composite resin with fibers can alter the fracture strength of teeth and may also be effective in reinforcing the weakened cusp in root canal treated teeth [13,14] which are in accordance with the study conducted by Bilgi et al. wherein the outcome of post-endodontic restorations in terms of fracture resistance was assessed and the highest fracture resistance was observed in the fiber-reinforced composite group when compared with other composites [15].

To overcome these problems, to some extent, various fiber-reinforced composites have been introduced. Fiber-reinforced composite restorations are resin-based restorations that contain fibers to improve the physical properties of the composites [16]. The fibers increase the structural properties of the material by acting as crack stoppers. The resin matrix protects the fibers and stabilizes their geometry to provide optimum reinforcement [16]. The everX Posterior is a premixed fiber-reinforced composite by GC consisting of E-glass fibers (9%) impregnated inside the nanohybrid composite. It is used as dentin replacement material in combination with an overlying enamel replacement composite [17]. This newly introduced material contains E-glass microfibers where an average size of the fiber is 140 µm and the diameter is 6 µm. It contains barium silicate glass filler particles with a size of 700 nm. It is claimed to have high wear resistance, excellent esthetics, and superior fracture toughness close to that of dentin due to the high number of short fibers strongly bonded to the resin matrix. Its thixotropic viscosity is said to have better adaptation property to the cavity floor without dipping, even when placed in upper molars. The launch of G-aenial universal injectable composite, which gives high strength, improved finishing, and esthetics that merge with teeth, was another development in composite materials.

The everX Posterior has already been used in the restoration of endodontically treated teeth, as a dentin replacement material, in combination with overlying solarX composite as enamel replacement material, but since everX Flow and G-aenial universal injectable are newly introduced materials and no clinical study has been conducted so far evaluating the clinical performance of these two materials, the present clinical study aimed to compare and evaluate the clinical performance of the combination of everX Flow with overlying G-aenial universal injectable with the combination of the everX Posterior with overlying solareX composite materials as post-endodontic class 1 restorations [17].

## 2. Materials and Methods

Ethical approval (*SVIEC/ON/Dent/BNPG19/D20010*) was taken from the Institutional Ethics committee and CTRI registration (*CTRI/2020/09/028127*) was completed before the commencement of the study. The sample description determined was based on the article by Ayna B, Elenk SC, Atakul F, Uysal E in 2009 [18].

The sample size has been obtained from the following formula:

Sample size N = CHISQUARE/W^2^

where W = 0.5; CHI SQUARE = 10; N = 10/(0.5)^2^ = 10/0.25 = 40; total sample size = 40; total number of groups = 2, and required sample size per group = 20.

The minimum sample size required was 40 (20 per group), but since it was an in vivo (clinical) study, 20% drop-out was expected and hence the total sample size was estimated to be around 50 (25 per group).The study was designed to follow the modified Declaration of Helsinki (2013) [19].

Inclusion criteria: The patients were between 18 to 60 years of age with permanent molar having class I defect (G. V. Black) which required primary endodontic treatment, and the tooth where the evaluation of the healing of periapical lesion was needed before proceeding with full coverage restoration were included in the study.

Exclusion criteria: Patients with poor oral hygiene, severe or chronic periodontitis, open apex, external or internal resorption, fractured or visibly cracked teeth, presence of parafunctional habits, severe attrition, malocclusion, tooth serving as an abutment, teeth with severe internal discoloration (tetracycline stain fluorosis), smokers, teeth having a remaining cavity wall thickness < 1 mm or with complete loss of the clinical crown, opposing ceramic full crown restoration, and absence of opposing tooth were excluded from the study.

Details of the materials used in the study are given in Table 1.

### 2.1. Endodontic Treatment Procedure 

Fifty patients (*n* = 25 in each group) having G. V. Black’s class I lesions in molars (maxillary and mandibular) requiring endodontic treatment followed by restoration were randomly selected based on the flip coin method. Isolation of the tooth was achieved with a rubber dam after the application of L.A. Access cavity was prepared following the principle of minimal invasive endodontics (i.e., conserving the sound tooth structure as much as possible). Cleaning and shaping were performed using the crown-down technique with rotary Ni-Ti files with mesial canals (MB and ML) in mandibular molars as well as buccal canals (MB and DB) in maxillary molars prepared up to 25/04 and palatal canals in maxillary molars, as well as distal canal/canals in mandibular molars which were prepared up to 30/04 along with standard irrigation protocol. Then obturation was carried out with a cold lateral compaction technique along with AH plus sealer. The entire treatment was performed by the principal investigator.

From here, the teeth were divided into two groups:

### 2.2. Group (A)—EverX Posterior Composite with Nanohybrid Solare-X Composite

**Figure 1:**
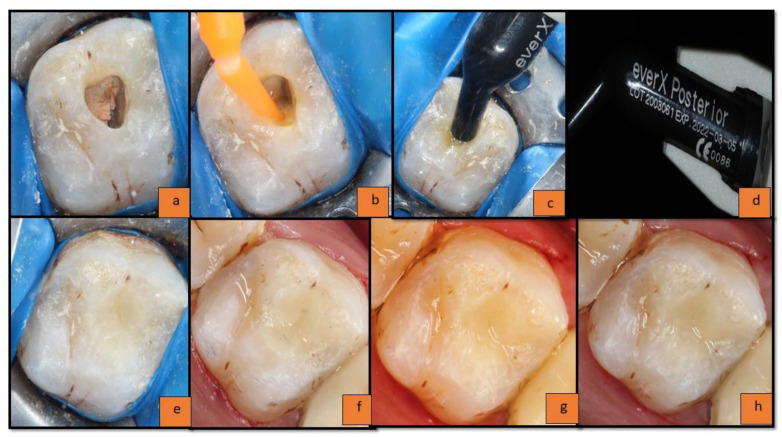
(**a**) Endodontically treated tooth; (**b**) application of universal bonding agent; (**c**) placement of everX posterior composite; (**d**) everX posterior composite material; (**e**) placement of solareX composite; (**f**) at baseline; (**g**) 6 months follow-up; (**h**) 1 year follow-up.

In the post-endodontic cavity (Figure 1a), a universal bonding agent (solareX universal bonding system, GC India Dental Pvt., Ltd., Telangana, India) was applied and air thinned until its milky appearance vanished, and it was light-cured (Woodpecker i-LED Plus Curing Light; Woodpecker Medical Instrument Co., Ltd., Guangxi, China) for 20 s with maximum intensity at 455 nm, light irradiance 1200 mW/cm^2^, according to the manufacturer’s instructions) (Figure 1b).

The restoration was started from the floor of the pulp chamber to an imaginary DEJ (leaving 2–2.5 mm thickness for enamel composite from the occlusal surface). The ‘incremental placement technique’ was used for the placement of substructure FRC material; everX Posterior (Figure 1c,d) (with the increment size up to an average of 4 mm since the maximum advisable increment to be used is up to 5 mm according to manufacturer’s instruction) followed by enamel replacement composite materials; solareX (with the increment size up to 2–2.5 mm) light-cured (Woodpecker i-LED Plus Curing Light; Woodpecker Medical Instrument Co., Ltd., Guangxi, China) for 20 s with maximum intensity at 455 nm, light irradiance 1200 mW/cm^2^, according to the manufacturer’s information) (Figure 1e). The sculpting was carried out using L.M. Arte instruments(India Viking ImpEx Pvt. Ltd., New Delhi, India). Restorations were then finished and polished as per the manufacturer’s instructions using the Shofu super snap mini kit (Shofu Dental India Pvt., Ltd., New Delhi, India). The immediate restoration (baseline) is presented in Figure 1f; follow-ups of the restoration are presented in Figure 1g (6 months) and Figure 1h (1 year).

### 2.3. Group (B): EverX 12 Composite with G-Aenial Universal Injectable Composite

**Figure 2:**
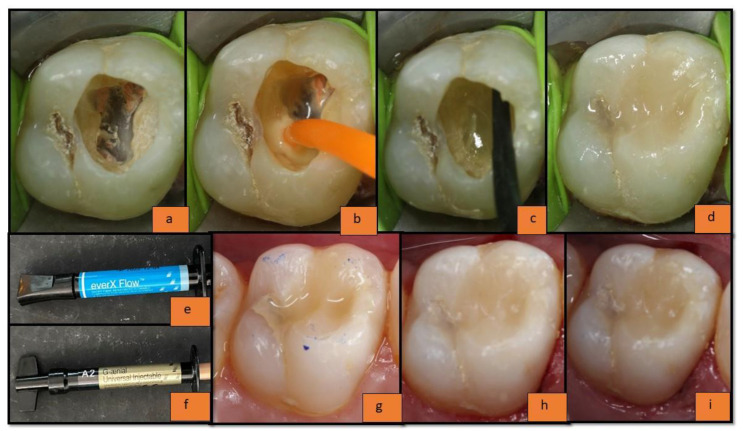
(**a**) Endodontically treated tooth; (**b**) application of universal bonding agent; (**c**) application of everX flow composite; (**d**) application of *g-aenial* universal injectable composite; (**e**) everX flow composite; (**f**) g-aenial universal injectable composite; (**g**) at baseline; (**h**) 6 months follow-up; (**i**) 1 year follow-up.

In the post-endodontic cavity (Figure 2a), a universal bonding agent was applied (solareX universal bonding system, GC India Dental Pvt., Ltd., Telangana, India) and air thinned until its milky appearance vanished, and it was light-cured (Woodpecker i-LED Plus Curing Light; Woodpecker Medical Instrument Co., Ltd., Guangxi, China) for 20 s with maximum intensity at 455 nm, light irradiance 1200 mW/cm^2^, according to the manufacturer’s instructions) (Figure 2b). The restoration was started from the floor of the pulp chamber to an imaginary DEJ (leaving 2–2.5 mm thickness for enamel composite from the occlusal surface). The ‘incremental placement technique’ was used for the placement of the substructure FRC material; everX flow (Figure 2c) (with the increment size up to an average of 4 mm since the maximum advisable increment to be used is up to 5 mm according to the manufacturer’s instruction) followed by enamel replacement composite materials; G-aenial universal injectable with the increment size up to 2–2.5 mm (Figure 2d–f). It was light-cured (Woodpecker i-LED Plus Curing Light; Woodpecker Medical Instrument Co., Ltd., Guangxi, China) for 20 s with maximum intensity at 455 nm, light irradiance 1200 mW/cm^2^, according to the manufacturer’s information). The sculpting was carried out using L.M. Arte instruments. Restorations were then finished and polished as per the manufacturer’s instructions of the Shofu super snap mini kit (Shofu Dental India Pvt., Ltd., New Delhi, India). The immediate restoration (baseline) is presented in Figure 2g; follow-ups of the restoration are presented in Figure 2h (6 months) and Figure 2i (1 year).

### 2.4. Evaluation of Restoration

All the restorations were clinically evaluated immediately after the restoration (to obtain the baseline data) (Figure 1f and Figure 2g), at 6 months (Figure 1g and Figure 2h), and at 1 year (follow-up data) (Figure 1h and Figure 2i) by a co-investigator; not by the principal investigator, based on the modified USPHS criteria—Cavo-surface Marginal Discoloration, Anatomic Contour, Marginal Integrity, Surface Texture, Fracture of Tooth, and Fracture of Restoration.

The co-investigator was not involved in the placement of the restorations and he/she was unaware of the materials used in this double-blinded study. All evaluations for both groups were carried out under a dental operating light using front-surface mouth mirrors and dental explorers.

The results obtained were tabulated and sent for statistical analysis wherein the *p*-value and chi-square value were calculated with SPSS software version 18.0.

## 3. Results

The statistical analysis using a Chi-square test showed no statistically significant difference in the clinical performance of group A—everX Posterior with a top layer of solarX Composite and group B—everX Flow with a top layer of G-aenial universal injectable composite in terms of Cavo-surface Marginal Discoloration, Anatomic Contour, Marginal Integrity, Surface Texture, Fracture of Tooth, and Fracture of Restoration at the end of baseline, at 6 months, and 1 year. The total number of samples lost to follow-up was 3 and 4 for group A and group B, respectively (Table 2).

### 3.1. Results of Individual USPHS Criteria

#### 3.1.1. Anatomic Contour 

In the present study, the normal anatomic form was observed at baseline in both groups. At 6 month intervals: in group A, 18 out of 24 samples showed normal anatomic form and hence received the Alpha score while 6 samples showed evident surface concavity and hence received the Bravo score. In group B, 20 out of 24 samples showed normal anatomic form while 4 samples showed evident surface concavity; hence received an Alpha score whereas at the end of 1 year, in group A, 12 out of 23 samples showed normal anatomic form and hence received Alpha score, while 11 samples showed evident surface concavity hence received Bravo score, and in group B, 15 out of 22 samples showed normal anatomic form; scored as Alpha, 5 samples showed evident surface concavity; scored as Bravo, 2 samples showed loss of restorative substance hence scored as Charlie because surface concavity was evident in these samples (Table 3). The comparative results for Anatomic Contour between group A and group B at the baseline, at 6 months, and at the 1 year interval were statistically non-significant. 

#### 3.1.2. Cavo-Surface Marginal Discoloration

In this study, there was no statistically significant difference between the two groups at any interval of time for both groups A and group B. In group A at baseline, all 25 samples received Alpha scores. At 6 months, in group A, 22 out of 24 samples received Alpha scores while 2 received Bravo scores. After 1 year, 23 restorations were present of which 17 received Alpha scores, 5 received Bravo scores, and 1 received Charlie scores. For group B, at 6 months, out of a total of 24 samples present, 20 received Alpha scores while 4 received Bravo scores. After 1 year, out of the 22 samples present, s15 received an Alpha score, 5 received a Bravo score, and 2 received a Charlie score (Table 3).

#### 3.1.3. Marginal Integrity 

For group A at baseline, out of 25 samples, all 25 samples received Alpha scores. At 6 months, out of the 24 samples present, 22 received an Alpha score while 2 received Bravo scores. After 1 year, out of the 23 samples present, 17 received an Alpha score, 5 received Bravo scores, and 1 received a Charlie score. For group B, at baseline, out of 25 samples, all 25 samples received Alpha scores. At 6 months, out of the 24 samples present, 20 received an Alpha score while 4 received a Bravo score. After 1 year, out of the 22 restorations present, 15 received an Alpha score while 5 received a Bravo score while 2 received a Charlie score. The present study showed that there was no statistically significant difference between the two groups at any interval of time (Table 3).

#### 3.1.4. Fracture of Restoration

In the present study, at the interval of 6 months, fracture of restoration was observed in 1 sample in group B and no fracture at all in Group A. At the end of 1 year, group A showed a fracture of restoration in 1 sample whereas, in group B, 3 samples showed a fracture of restoration. Statistically, the difference in both the groups was non-significant (Table 3).

#### 3.1.5. Fracture of Tooth

In the present study, a fracture of the tooth was observed in 1 sample at the interval of 6 months in both groups. Whereas at the end of 1 year, fracture of the tooth was observed in 1 and 2 samples in group A and group B, respectively (Table 3). Again, the difference in both groups was found to be statistically non-significant. 

#### 3.1.6. Surface Texture

For group A, at baseline, out of 25 samples, 24 samples received Alpha scores while 1 received Bravo. At 6 months, out of the 24 restorations present, 21 received Alpha scores, 2 received Bravo scores, and 1 received a Charlie score. After 1 year, out of the 23 restorations present, 7 received an Alpha score while 16 received a Bravo score. For group B, at baseline, out of 25 samples, all 25 samples received Alpha scores. At 6 months, out of the 24 samples present, 19 received Alpha scores, 4 received Bravo scores while 1 received a Charlie score. After 1 year, out of the 22 samples present, 10 received Alpha scores, 11 received Bravo scores, and 1 received a Charlie score (Table 3). The present study showed a statistically non-significant difference in both groups.

## 4. Discussion

In the past, many in vitro studies on the fiber-reinforced composite have been conducted by various researchers showing an enhancement in the fracture strength of root canal-treated teeth [4,7] However, several clinical studies evaluating the reinforcing effect of fiber-reinforced composite on endodontically treated teeth is still scarce. Moreover, no studies are available on comparing the clinical performance of the combination of everX Posterior with solareX and everX *Flow* with G-aenial universal injectable composite since these are recently introduced materials. 

In the current study, modified USPHS criteria were used for clinical evaluation of fiber-reinforced composites as post-endodontic class I restorations in molars which has a high-reliability rate at the baseline, at 6 months, and at the 1 year time intervals [7].

### 4.1. Anatomic Contour 

The comparative results for Anatomic Contour between group A and group B at the baseline, at 6 months, and at the 1 year interval were statistically non-significant. The possible reason for this could be the use of a nano-hybrid composite (solareX) in group A and *G-aenial universal injectable* in group B. Nano-hybrid composites are claimed to have nanofillers, glass fillers, and pre-polymerized fillers resulting in low polymerization shrinkage, adequate flow, and better adaptation to the cavity walls. This explains the clinically acceptable performance of group A restorations. For group B, G-aenial universal injectable was used which is a newly introduced composite and is based on full coverage silane coating (FSC) technology which makes it an efficient enamel replacement material, not requiring the need for any overlying composite. Additionally, it does have a high thixotropic viscosity resulting in better adaptability and no slumping of the material and this could be the reason it provided long-lasting superior anatomic contour in a majority of its samples. Moreover, in both the groups, the incremental layering technique and proper sculpting were conducted with L.M. Arte instruments which again helped in achieving proper anatomic contour.

### 4.2. Cavo-Surface Marginal Discoloration

Marginal discoloration is known to be a very important element of a restorative procedure since the problem in the tooth-restoration interface like marginal staining is related to material physics and mechanical properties like modulus of elasticity, coefficient of thermal expansion, curing shrinkage and, most importantly, to enamel margin finishing [20]. Overall, the clinical performance of both groups was satisfactory. These results are contradictory to the study conducted by Robert et al. on the clinical evaluation of nanohybrid composite restoration on a posterior tooth, wherein a gradual discoloration of composite resin was noticed over a while [21]. The long-term performance of restoration may also depend on the hydrophilicity and solvent type of the adhesive system used under the restorative material [22]. These parameters may promote the degradation of the bond, leading to further marginal discoloration and secondary caries. In the present study, the solareX universal bonding system was used; this could be the reason for less marginal discoloration. Another important reason for not having prominent marginal discoloration in most of the samples of both groups was the removal of the oxygen inhibition layer at the time of restoration. Moreover, to some extent, the improved filler technology, and modified organic matrices of both solareX and G-aenial universal injectable composites offer a greater degree of polymerization, reducing the microleakage at the restoration tooth interface which might have further improved the clinical performance in both the groups in terms of marginal discoloration.

### 4.3. Marginal Integrity

The marginal integrity can be attributed to the properties of both the enamel replacing materials: solareX and G-aenial universal injectable composites. Since solareX is said to have an optimized resin formulation, and new generation pre-polymerized fillers and G-aenial universal injectable, as mentioned earlier, are said to have FSC technology with its short fibers pre-incorporated in resin rendering it a stronger material, there would have been less polymerization shrinkage and proper adaptation of the restorative material to the cavity walls maintaining the marginal integrity in most of the samples in both the groups.

### 4.4. Fracture of Restoration and Tooth

Fracture of restoration and fracture of the tooth, in previous literature, has stated that the fibers can withstand stress and crack propagation due to high tensile strength, density, and percentage elongation [23]. Additionally, E-glass fibers are less rigid compared to previous glass fibers and can be easily adapted closely to the teeth [24]. The direction and the orientation of the fibers in the resin matrix are also important factors in terms of reinforcing the composite material. According to the Krenchel factor, E-glass fibers combined with resin matrix offer an isotropic strengthening effect in several directions, not just one or two. This might be the reason for increasing the fracture strength of both groups [25]. The results of the present study are as per the study conducted by Lutharia et al., wherein the fracture resistance of endodontically treated MOD maxillary premolars restored with either composite resin or FRC with different types of fibers was assessed. The results showed that the highest fracture resistance was shown by the composite reinforced with impregnated glass fibers compared to other groups [7].

A previous study showed that when a short fiber-reinforced composite was used as a substructure under a particulate filler composite, the load-bearing capacity of this combination increased linearly as the thickness of the layer of FRC increased in endodontically treated teeth [26]. Additionally, in an in vitro study conducted by Eapen et al., the short glass fibers composite used as a base was covered with only a 1 mm thick layer of nano-hybrid composite and showed increased fracture toughness similar to the negative control group [27]. In the present study, both the FRCs (everX Posterior and everX flow) were having an average thickness of 4–5 mm as substructure materials covered by up to a 2 to 2.5 mm thick layer of solareX composite and G-aenial universal injectable composites, respectively. This might have provided the clinically satisfactory and acceptable results in both the groups.

Another important factor is the ‘critical fiber length’, which is the minimum length at which the center of the fiber reaches its ultimate tensile strength when the matrix reaches its maximum shear strength. According to the literature [28], the minimum length should be 0.5 to 1.6 mm to show improved properties. Since E-glass fibers have an average length of 3 mm, this could be the reason that both the FRCs in the present study showed satisfactory as well as comparable clinical performance. 

EverX Posterior has already been reported to have superior physical properties compared with conventional composites and it is recommended for use in high stress-bearing application areas by Garoushi et al. Since everX flow is a new material, based on the results of the present clinical study, it can be stated that it performed well as a class I post-endodontic restoration which was statistically equivalent to the everX Posterior. Still, further clinical studies could be conducted to validate these results.

Discussing the possible whys and wherefores for the fracture of the teeth in the present study that was observed in 3 samples in both groups for 1 year, there may be a variety of reasons that might have caused the fracture. Significant ones are:

1. Patient’s habits: If the patient had a habit of chewing betel nuts (or any other similar habit) or had hard consistency food in his routine diet; non-veg, seafood, etc. This might have caused cracks in the restoration leading to a complete fracture over 1 year.
2. Location of the tooth in the arch: If there was any occlusal discrepancy already existing or developed later due to some other reasons, this might have shifted the occlusal forces to the tooth restored with FRC in this experimental study, leading to a possible fracture.
3. Poor oral hygiene: Composite materials require good oral hygiene for their longevity in clinical conditions as the accumulation of plaque and subsequent microleakage around them might lead to various complications including the fracture of the restoration in the long run.

### 4.5. Surface Texture

The present study showed a statistically non-significant difference in both groups. The degree of conversion, finishing, and polishing procedures, as well as the composition of material, organic matrix, inorganic filler, etc., are the factors that can affect the surface quality of composite resins [28]. As mentioned earlier in this discussion, the improved filler technology in the nanohybrid composite (solareX) and SFC technology in the G-aenial universal injectable composite might be the reason for having a clinically sustainable surface texture up to 1 year in follow-up in most of the samples in both the groups.

## 5. Limitations of the Present Study

Teeth selected in the study were deep class I lesions requiring endodontic treatment (occlusal surface with both the marginal ridges intact). Hence, clinical evaluation of the badly mutilated teeth involving the proximal margins restored with FRC is required. Sample size, as well as follow-up time duration taken in the present study, was relatively small. Hence, clinical studies with a larger sample size and long-term follow-up are required for further acceptable and reliable results.

## 6. Conclusions

Within the limitations of the present study, based on 1 year of clinical performance (according to modified USPHS criteria), it could be concluded that:

1. Clinical performance of the combination of everX Flow with overlying G-aenial Universal Injectable Composite proved to be comparable with everX Posterior with overlying solareX composite as post-endodontic restorations in class I lesions in molars without having a statistically significant difference.
2. Individually, the clinical performance of newly introduced materials such as everX Flow (as a substrate dentin replacement material) and G-aenial Universal Injectable Composite (as enamel replacement material) proved to be similar compared with their experimental counterparts, everX posterior and solareX Composite materials.
3. Bilayer restorations, a combination of new dentin replacement material with overlying new enamel replacement materials with inherent advanced properties, can mimic the behavior of natural tooth tissues evidencing more conservative, less invasive, and long-lasting restorations, especially in endodontically treated teeth.

## Figures and Tables

**Table 1:** Details of the materials used in this study.

Product Name	Manufacturer	Components	Batch	Expiry	Shade
Solare Universal Bond	GC India Dental Pvt. Ltd. Telangana, India	4-MET, MDP, MDTP, Photoinitiator, Nano filler	2007011	June 2022	-
everX Posterior	GC Corporation Tokyo, Japan	Bis-GMA, PMMA, TEGDMA, Salinated E-glass Fiber, Barium Glass	2003061	March 2022	-
everX Flow	GC Corporation Tokyo, Japan	Bis—MEPP, TEGDMA, UDMA, E-glass Fiber, Barium Glass, Silicon Dioxide	1912051	December 2022	-
G-aenial universal injectable	GC Corporation Tokyo, Japan	UDMA, Bis-MEPP, TEGDMA, Fillers: SiO_2_, Barium Glass	1909051	September 2022	A2

**Table 2:** Samples lost to follow-up in group A and B at different time intervals.

GROUP	Time Interval	Samples Present (No.)	Samples Lost to Follow-Up (No.)	Samples Lost to Follow-Up (%)
group A	At Baseline	25	0	0%
	At 6 months	24	1	4%
	At 1 year	23	2	8%
group B	At Baseline	25	0	0%
	At 6 months	24	1	4%
	At 1 year	22	3	12%

**Table 3:** Results of individual USPHS criteria.

Anatomic Contour	Baseline		6 Months	1 Year		
	group A (*n* = 25)	group B (*n* = 25)	group A (*n* = 24)	group B (*n* = 24)	group A (*n* = 23)	group B (*n* = 22)
Alpha	25 (100%)	25 (100%)	18 (75%)	20 (83.3%)	12 (52.2%)	15 (68.2%)
Bravo	0 (0%)	0 (0%)	6 (25%)	4 (16.7%)	11 (47.8%)	5 (22.7%)
Charlie	0 (0%)	0 (0%)	0 (0%)	0 (0%)	0 (0%)	2 (9.1%)
	NA		Chi-square = 0.505, Exact *p*-value = 0.724	Chi-square = 4.563, Exact *p*-value = 0.091		
**Cavo-Surface Marginal Discoloration**	**Baseline**		**6 Months**	**1 Year**		
	group A (*n* = 25)	group B (*n* = 25)	group A (*n* = 24)	group B (*n* = 24)	group A (*n* = 23)	group B (*n* = 22)
Alpha	25 (100%)	25 (100%)	22 (91.7%)	20 (83.3%)	17 (73.9%)	15 (68.2%)
Bravo	0 (0%)	0 (0%)	2 (8.3%)	4 (16.7%)	5 (21.7%)	5 (22.7%)
Charlie	0 (0%)	0 (0%)	0 (0%)	0 (0%)	1 (4.3%)	2 (9.1%)
	NA		Chi-square = 0.762, Exact *p*-value = 0.666	Chi-square = 0.436, Exact *p*-value = 0.890		
**Marginal Integrity**	**Baseline**		**6 Months**	**1 Year**		
	group A (*n* = 25)	group B (*n* = 25)	group A (*n* = 24)	group B (*n* = 24)	group A (*n* = 23)	group B (*n* = 22)
Alpha	25 (100%)	25 (100%)	22 (91.7%)	20 (83.3%)	17 (73.9%)	15 (68.2%)
Bravo	0 (0%)	0 (0%)	2 (8.3%)	4 (16.7%)	5 (21.7%)	5 (22.7%)
Charlie	0 (0%)	0 (0%)	0 (0%)	0 (0%)	1 (4.3%)	2 (9.1%)
	NA		Chi-square = 0.762, Exact *p*-value = 0.666	Chi-square = 0.436, Exact *p*-value = 0.890		
**Fracture of Restoration**	**Baseline**		**6 Months**	**1 Year**		
	group A (*n* = 25)	group B (*n* = 25)	group A (*n* = 24)	group B (*n* = 24)	group A (*n* = 23)	group B (*n* = 22)
Alpha	25 (100%)	25 (100%)	24 (100%)	23 (95.8%)	22 (95.7%)	19 (86.4%)
Bravo	0 (0%)	0 (0%)	0 (0%)	1 (4.2%)	1 (4.3%)	3 (13.6%)
Charlie	0 (0%)	0 (0%)	0 (0%)	0 (0%)	0 (0%)	0 (0%)
	NA		Chi-square = 1.021, Exact *p*-value = 1.000	Chi-square = 1.198, Exact *p*-value = 0.346		
**Tooth Fracture**	**Baseline**		**6 Months**	**1 Year**		
	group A (*n* = 25)	group B (*n* = 25)	group A (*n* = 24)	group B (*n* = 24)	group A (*n* = 23)	group B (*n* = 22)
Alpha	25 (100%)	25 (100%)	23 (95.8%)	23 (95.8%)	22 (95.7%)	20 (90.9%)
Bravo	0 (0%)	0 (0%)	1 (4.2%)	1 (4.2%)	1 (4.3%)	2 (9.1%)
Charlie	0 (0%)	0 (0%)	0 (0%)	0 (0%)	0 (0%)	0 (0%)
	NA		Chi-square = 0, Exact *p*-value = 1.000	Chi-square = 0.407, Exact *p*-value = 0.608		
**Surface Texture**	**Baseline**		**6 Months**	**1 Year**		
	group A (*n* = 25)	group B (*n* = 25)	group A (*n* = 24)	group B (*n* = 24)	group A (*n* = 23)	group B (*n* = 22)
Alpha	24 (96%)	25 (100%)	21 (87.5%)	19 (79.2%)	7 (30.4%)	10 (45.5%)
Bravo	1 (4%)	0 (0%)	2 (8.3%)	4 (16.7%)	16 (69.6%)	11 (50%)
Charlie	0 (0%)	0 (0%)	1 (4.2%)	1 (4.2%)	0 (0%)	1 (4.5%)
	Chi-square = 1.020, Exact *p*-value = 1.000		Chi-square = 0.767, Exact *p*-value = 0.829	Chi-square = 2.434, Exact *p*-value = 0.283		

## Data Availability

Not applicable.
